# Miscellaneous Notices

**Published:** 1843-03

**Authors:** 


### Miscellaneous Notices.

*Dr. Maynard's Drill-Stock.*—We took occasion, in the first number of the present volume of the Journal, to state, that the drill-stock submitted to the last meeting of the American Society of Dental Surgeons, by Dr. E. Maynard, of Washington City, received the unanimous approval of its members. Since which time, through the kindness of Dr. M., who kindly presented us with one, we have had an opportunity of testing its value, and we are happy to say, that the favourable opinion which we then expressed with regard to its merits, has been fully confirmed. There are but few cases, it is true, in which it can be advantageously employed, but the amount of labour and trouble saved even in these, is more than sufficient to compensate for its cost.

*Lancing the Gums in Stridulous Convulsions.—To the Editor*—Sir: nearly four months ago you published a statement of mine describing the treatment adopted by Dr. Marshall Hall in a case of stridulous convulsions occurring in one of my children. You also published a former statement in which I endeavored to show that nearly all my children have been affected, more or less, with the same complaint, which had baffled the skill of all the medical men whom I had consulted on the subject, and had proved fatal to three of my family.

The means resorted to by Dr. Hall in my last child were fully explained by himself in The Lancet of the 9th July, and since commented upon by several correspondents, and, in particular, the frequent lancing of the gums. I cannot discuss the subject with professional men, but for the benefit of other children who may be afflicted like mine, I think it right to make known that the plan proposed by Dr. Hall has been persevered in with the child up to the present time (altogether during six months), and with eminent success. The child has had no return whatever of the crowing spasms, has grown healthy, has a mouthful of teeth, runs alone, and though not a large or robust child, is every thing else we could wish. He is still supported entirely at the breast. The gums continue to be lanced occasionally, and though the operation is always accompanied with cries, smiles soon follow, showing that the inconvenience is but temporary, and, with all affection for my children, I would rather have his gums lanced every day of his infantile life, than expose him to a return of one of those dreadful spasms and destructive convulsions which I have so often unhappily witnessed as the result of the complaint in question, and I am happy to express my gratitude to Dr. Hall for having found a successful mode of treatment for that which I had been led to believe, and experience had almost convinced me, was irremediable. I am, sir, your obedient servant. W. H. Grey.

South Lambeth, Nov. 1, 1842. [*London Lancet.*

In our last number we copied an article from "The American Journal of Medical Sciences," describing an operation performed by Dr. C. B. Gibson, on Moses Lee, for Osteo-Sarcoma of the Lower Jaw, without the proper credit. We now with great pleasure correct our error, and acknowledge our indebtedness to the above excellent Journal for the statement of the case.

